# Functional expression and characterization of five wax ester synthases in *Saccharomyces cerevisiae *and their utility for biodiesel production

**DOI:** 10.1186/1754-6834-5-7

**Published:** 2012-02-24

**Authors:** Shuobo Shi, Juan Octavio Valle-Rodríguez, Sakda Khoomrung, Verena Siewers, Jens Nielsen

**Affiliations:** 1 Department of Chemical and Biological Engineering, Chalmers University of Technology, Kemivägen 10, SE 412 96, Göteborg, Sweden

**Keywords:** Biodiesel, fatty acid ethyl esters, metabolic engineering, *Saccharomyces cerevisiae*, wax ester synthase

## Abstract

**Background:**

Wax ester synthases (WSs) can synthesize wax esters from alcohols and fatty acyl coenzyme A thioesters. The knowledge of the preferred substrates for each WS allows the use of yeast cells for the production of wax esters that are high-value materials and can be used in a variety of industrial applications. The products of WSs include fatty acid ethyl esters, which can be directly used as biodiesel.

**Results:**

Here, heterologous WSs derived from five different organisms were successfully expressed and evaluated for their substrate preference in *Saccharomyces cerevisiae*. We investigated the potential of the different WSs for biodiesel (that is, fatty acid ethyl esters) production in *S. cerevisiae*. All investigated WSs, from *Acinetobacter baylyi *ADP1, *Marinobacter hydrocarbonoclasticus *DSM 8798, *Rhodococcus opacus *PD630, *Mus musculus *C57BL/6 and *Psychrobacter arcticus *273-4, have different substrate specificities, but they can all lead to the formation of biodiesel. The best biodiesel producing strain was found to be the one expressing WS from *M. hydrocarbonoclasticus *DSM 8798 that resulted in a biodiesel titer of 6.3 mg/L. To further enhance biodiesel production, acetyl coenzyme A carboxylase was up-regulated, which resulted in a 30% increase in biodiesel production.

**Conclusions:**

Five WSs from different species were functionally expressed and their substrate preference characterized in *S. cerevisiae*, thus constructing cell factories for the production of specific kinds of wax ester. WS from *M. hydrocarbonoclasticus *showed the highest preference for ethanol compared to the other WSs, and could permit the engineered *S. cerevisiae *to produce biodiesel.

## Background

Natural wax esters are typically esters of long-chain fatty acids and long-chain alcohols [1]; due to their special properties, they have been widely used in lubricants, cosmetics, linoleum, printing inks, candles and polishes. For example, wax esters consisting of fatty acids with 20 carbon atoms (C_20_) and C_20 _alcohols are outstanding lubricants [2]; wax esters consisting of C_14 _to C_20 _fatty acids and a C_2 _alcohol represent good diesel fuels [3]. Today, wax esters are harvested from plants and animal tissues, or generated by chemical synthesis using fossil sources, and this is considered to be the main limitation for their application due to the restricted availability and high costs of existing sources [2,4]. Thus, there is a strong demand for the development of an alternative bioprocess to obtain cheap and sustainable wax esters.

Wax ester synthases (WSs) are promiscuous enzymes involved in wax ester synthesis from alcohols and acyl coenzyme As (CoAs) [5]. Various WSs have different preferences for substrates with varied chain length and their unspecificity has been used in several biotechnological applications for ester production, for example, jojoba-like wax esters and fatty acid ethyl esters. In general, WSs naturally accept acyl groups with carbon chain lengths of C_16 _or C_18 _and linear alcohols with carbon chain lengths ranging from C_12 _to C_20_. Reported activities of WSs with short-chain alcohols were low [6]. Depending on the substrate specificity of the WS, various mixtures of wax esters with specific chain-length composition can be generated, which have optimal chemical compositions for certain specialty markets. Designed wax ester mixtures that do not normally exist in nature can be generated by expressing mutant WSs or a combination of WSs. It has become feasible to produce biotechnological wax esters in bioreactors [2,4,5,7-10]. The types of wax esters synthesized by a specific WS are determined by the WS substrate preference and available substrates provided through the host's metabolism.

One of the best characterized cell factories is the eukaryotic model organism, *Saccharomyces cerevisiae*. The well-studied industrial microorganism *S. cerevisiae *offers a number of advantages for producing fatty acid derived products (for example, wax esters) due to the ease of cultivation and genetic manipulation, its short generation time and extensive knowledge about its fatty acid metabolism [11-14]. The development of *S. cerevisiae *as a cell factory would represent a good choice for wax ester production.

There are three unrelated families of WSs found in higher plants, mammals and bacteria [2]. The first identified WS of plants, jojoba embryo wax ester synthase, did not show activity when heterologously expressed in *S. cerevisiae *[10]. The second group of identified WSs was from bacteria identified by a homology search using the jojoba WS amino acid sequence. Some of the WSs from bacteria are bifunctional enzymes, that is, they functions as WS and as acyl-CoA:diacylglycerol acyltransferase (DGAT). The third group of WSs is from mammals, for example, WS from *Mus musculus *C57BL/6 [15]. WSs from different organisms represent candidates for ester synthases with various substrate and product chain-length preferences. Some of these WSs have been expressed in *Escherichia coli*, but significant differences in substrate specificities were observed, depending on whether yeast or *E. coli *was used as the host for heterologous expression [9,10].

We therefore decided to conduct a comparison of the substrate preferences of the representative WSs from bacteria and mammals in *S. cerevisiae*, where little information on WS expression and substrate preference is available. WS/DGAT from *Acinetobacter baylyi *ADP1 has been adopted in *E. coli *for ester production [4,8,16-18]. WS homologs are frequently found in the genomes of actinomycetes such as *Rhodococcus *[19,20] or in the genome databases of several marine bacteria like *Marinobacter *[19,21] and *Psychrobacter *[19]. Few reports are available about WS from mammals. Recently, a study reported the isolation and characterization of a wax synthase enzyme from *Mus musculus *C57BL/6, which was expressed in human embryonic kidney (HEK) 293 cells [15]. Therefore, five different WSs from *A. baylyi *ADP1, *Marinobacter hydrocarbonoclasticus *DSM 8798, *Rhodococcus opacus *PD630, *M. musculus *C57BL/6 and *Psychrobacter arcticus *273-4 were chosen and characterized in *S. cerevisiae*, which is being considered as a platform for ester production. Apart from the *A. baylyi *WS, this is the first scientific study that has demonstrated WS activity from the other four species in *S. cerevisiae*. Variations in the substrate preferences of the WSs would lead to differences in the chain-length composition of products with various specialty applications.

A process that utilizes the promiscuous activity of WS is the biosynthesis of biodiesel. Biodiesel mixtures are composed of linear fatty acid methyl esters or fatty acid ethyl esters (FAEEs) ranging from C_8 _to C_22_, but are usually dominated by chain lengths from 16 to 18 carbons, for example, C_16 _to C_18 _methyl or ethyl esters [3]. Biodiesel, currently derived from plant oil, is already produced in an increasing number of countries and has been considered as a clean and sustainable liquid fuel alternative to fossil fuels [22,23]. However, the high costs and limited availability of plant oils are becoming a problem for large-scale commercial viability of biodiesel production, and different ways have been explored to address this problem [23,24].

**Figure 1:**
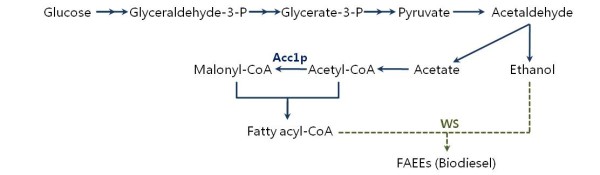
**Fatty acid ethyl esters biosynthesis involving heterologous wax ester synthases in *Saccharomyces cerevisiae***. Single and double arrows represent single and multiple enzymatic steps; dashed arrows represent heterologous pathways. WS: wax ester synthase; Acc1p: acetyl-CoA carboxylase.

As shown in Figure 1, WS can catalyze the formation of FAEEs (as in biodiesel) from ethanol and fatty acyl-CoA, and here this strategy was used to construct proof of principle biodiesel microbial cell factories that could ultimately form the basis for large-scale commercial biodiesel production and result in a fully sustainable fuel [3,16,23]. Previously reported renewable diesel cell factories have been mainly developed in the model organism *E. coli*, by expressing WS from *A. baylyi *ADP1, a bifunctional enzyme functioning as WS and DGAT and encoded by the gene a*tfA *[8,16-18]. However, the activity of WS from *A. baylyi *ADP1 for short-chain alcohols and the ability to form biodiesel is poor, which is one of the major limitations for biodiesel production. A more suitable WS that has higher activity for producing biodiesel was chosen based on the evaluation of five WSs in yeast. Furthermore, compared to the previously used *E. coli *system, yeast itself has the ability to produce higher amounts of ethanol, one of the two substrates needed for *de novo *production of biodiesel from glucose, which makes it a more suitable host for this process. Besides evaluating WSs in yeast, we also demonstrated that it is possible to redirect more flux towards biodiesel production through engineering of the lipid metabolism. As shown in Figure 1, acetyl-CoA carboxylase (Acc1p) catalyzes the formation of malonyl-CoA and is often ascribed to be crucial for enhancing synthesis of fatty acyl-CoAs [11,12]. We therefore also evaluated the effect of over-expressing Acc1p in a WS-expressing strain.

## Results and discussion

### Functional expression and characterization of five wax ester synthases in yeast

Low WS activity in crude cell extracts of *S. cerevisiae *has been reported. However, no formation of wax esters was detected *in vivo *[9]. Only heterologous expression of the *A. baylyi *bifunctional WS/DGAT enzyme could result in the formation of detectable amounts of wax esters.

Several WSs have been identified in bacteria and mammals [5,15,19-21], out of which we selected five WSs for expression and characterization in *S. cerevisiae *CEN.PK 113-5D under control of the strong *TEF1 *promoter using plasmid pSP-GM2 [25]. The selected five WSs are from *A. baylyi *ADP1, *M. hydrocarbonoclasticus *DSM 8798, *R. opacus *PD630, *Mus musculus *C57BL/6 and *P. arcticus *273-4. In an effort to increase expression of the WSs, the gene sequences were codon optimized for expression in *S. cerevisiae*. Their transcription in *S. cerevisiae *CEN.PK 113-5D was initially confirmed by reverse transcription PCR (data not shown).

As described above, WSs have the capability of catalyzing the esterification of linear alcohols with saturated or unsaturated acyl-CoA thioesters. However, to date, little is known about the substrate specificity of WSs and some WSs did not even possess significant WS activity when expressed in recombinant *S. cerevisiae *[10]. Here, we tested the activity of the five WSs with alcohols ranging from ethanol to octadecanol using^14^C-labeled palmitoyl-CoA as the acyl donor. Table 1 summarizes the results of the enzyme activity analysis that was performed *in vitro *using crude cell extracts and^14^C-labeled substrate. A low WS activity was detected in cell extracts derived from the negative control *S. cerevisiae *CB0. Extracts of the strains that expressed different WSs, *S. cerevisiae *CB1 (expressing the WS from *A. baylyi *ADP1), CB2 (expressing the WS from *M. hydrocarbonoclasticus *DSM 8798), CB3 (expressing the WS from *R. opacus *PD630), CB4 (expressing the WS from *Mus musculus *C57BL/6), and CB5 (expressing the WS from *P. arcticus *273-4), showed different activity to alcohols with varied chain length, from C_2 _to C_18_, as the substrates, which was significantly higher than the background levels. The results of these measurements allowed a direct comparison of the substrate preferences of the selected WSs in yeast since substrate specificities may vary with the host organism used for heterologous expression [9].

**Table 1:** Comparison of acyl acceptor specificities of different wax ester synthases using palmitoyl coenzyme A as the acyl donor

Acyl acceptor	Wax ester synthase activity (pmol/mg protein/min)					
	
	CB0	CB1	CB2	CB3	CB4	CB5
Ethanol	0.67 ± 0.15	4.6 ± 0.55	8.1 ± 1.87	2.7 ± 0.37	3.8 ± 0.51	5.9 ± 0.83
Butanol	0.42 ± 0.21	10.8 ± 1.60	14.6 ± 1.75	6.8 ± 0.82	3.5 ± 0.53	4.2 ± 0.46
1-hexanol	0.72 ± 0.20	17.3 ± 2.04	33.8 ± 3.77	16.1 ± 2.29	10.2 ± 1. 59	18.7 ± 2.19
1-octanol	0.83 ± 0.19	23.0 ± 2.39	45.7 ± 4.51	32.3 ± 3.84	22.3 ± 2.44	17.7 ± 1.67
1-decanol	0.75 ± 0.21	19.7 ± 3.11	41.1 ± 4.13	37.3 ± 3.90	33.5 ± 2.22	27.5 ± 2.50
1-dodecanol	0.78 ± 0.17	31.8 ± 3.48	48.4 ± 4.56	36.7 ± 3.78	44.2 ± 3.07	42.8 ± 3.11
1-tetradecanol	0.81 ± 0.27	45.0 ± 4.72	49.7 ± 4.38	33.5 ± 3.66	35.1 ± 2.87	36.5 ± 3.03
1-hexadecanol	0.90 ± 0.20	41.6 ± 2.21	49.0 ± 3.65	28.9 ± 3.29	35.5 ± 2.91	39.1 ± 2.72
1-octadecanol	0.77 ± 0.21	43.6 ± 2.21	40.1 ± 3.77	30.9 ± 3.12	39.5 ± 2.64	38.3 ± 2.32

Values are the mean of at least three independent assays ± standard deviation.

WS from *A. baylyi *ADP1 exhibited a broad substrate range that included linear alcohols from C_2 _to C_18 _(Table 1). It showed the highest activity towards long-chain alcohols (C_12 _to C_18_) and the activity decreased gradually from C_12 _to C_2_. The WS activity on 1-hexadecanol was slightly lower than according to a previous study, where the same WS was expressed in *S. cerevisiae *H1246 [9] using a different expression system, but here we were able to demonstrate its activity on an extended substrate range. The previous report showed that heterologous expression of WS from *A. baylyi *ADP1, which also has DGAT activity [4,9], could confer neutral lipid (triacylglycerol (TAG) and wax ester) biosynthetic ability to the host strain, the quadruple mutant *S. cerevisiae *H1246 (d*ga1*, l*ro1*, a*re1*, a*re2*).

WS from *M. hydrocarbonoclasticus *DSM 8798 exhibited a lower specificity compared to the other WSs when expressed in *S. cerevisiae *(Table 1). It showed similar activity towards alcohols from C_6 _to C_18_, which is in accordance with a previous study that reported similar activity of the purified protein on C_10 _to C_16 _alcohols together with palmitoyl-CoA, albeit with a lower activity on octadecanol [21]. Due to its broader activity, cell extracts from strains expressing this enzyme not only showed a higher performance with long- and medium-chain alcohols ranging from C_6 _to C_18_, but also with the short chain alcohols ethanol and butanol compared to strains expressing any of the other WSs, which makes this WS a good candidate for the production of fatty acid esters from short-chain alcohols.

WS from *R. opacus *PD630 showed a similar activity towards alcohols from C_8 _to C_18_, and there was a substantial drop in activity when the alcohol chain length was decreased from C_8 _to C_6 _(Table 1). This enzyme has also been expressed in *E. coli *and has shown low DGAT activity [20]. However, no preference for specific acyl-CoA or alcohol substrates was reported in that study. In *E. coli*, the enzyme activity was measured using [1-^14^C] palmitoyl-CoA and 1-hexadecanol [20] and found to be 4.65 ± 0.04 pmol/mg protein/min, which is clearly lower than the activity found here in *S. cerevisiae*, which was 28.9 ± 3.29 pmol/mg protein/min (Table 1).

WS from *M. musculus *C57BL/6 also exhibited a broad substrate range (Table 1). It showed the highest activity towards long- and medium-chain length alcohols (C_10 _to C_18_), and the highest activity for C_12 _alcohol. This enzyme has been expressed in HEK 293 cells, which showed little or no DGAT activity and also showed greatest WS activity with alcohols from C_10 _to C_18 _[15]. Here we have shown the successful construction of the mammalian wax esters biosynthetic pathway in a microorganism for the first time, which demonstrates the feasibility of adopting mammalian wax esters in industrial microorganisms.

WS from *P. arcticus *273-4 has 50.4% identity to the WS from *A. baylyi *ADP1 [19], and has not been characterized before. In this study, its WS activity was confirmed in *S. cerevisiae *for the first time. Activity with alcohols from C_12 _to C_18 _was relatively high, with a drop in activity when the alcohol chain length decreased to 10 carbons. However, whether this enzyme exhibits DGAT activity remains to be elucidated. *P. arcticus *273-4 has remarkable activity levels in low temperatures, even at temperatures below 0°C [26]. It has been reported that lowering the growth temperature of batch cultures of *S. cerevisiae *results in increased levels of lipids [27] and this enzyme may be useful in this context.

All expressed WSs displayed a general preference for long-chain alcohols and a lower activity for short-chain alcohols, and the specific substrate preference varied among different WSs. Our findings have clearly shown the chain-length specificities and the broad substrate range of the different WSs. It will be possible to harness WS diversity for biotechnological production of individual esters and/or mixtures of esters with a particular combination of chain-length distribution that do not normally occur in nature in a single species. These rationally designed wax ester biosynthetic pathways can then be introduced into *S. cerevisiae *for industrial production of specific wax esters.

### Application of wax ester synthases for biodiesel production

Biodiesel, the primary renewable alternative to petroleum-based diesel fuel, is composed of fatty acid methyl and ethyl esters. The substrate profiles in Table 1 show that all WSs are able to catalyze the esterification of a fatty acid to ethanol to synthesize FAEEs (such as biodiesel). *S. cerevisiae *is already a good ethanol producer and produces fatty acids with a chain length of mainly 16 or 18 carbon atoms [12], indicating its ability to provide the required two substrates for WS. As described above, the WSs have the capability to accept ethanol as a substrate, resulting in the formation of FAEEs. Therefore, a *de novo *biodiesel (FAEEs) biosynthesis process can be established by introducing WS in *S. cerevisiae *[9].

**Figure 2:**
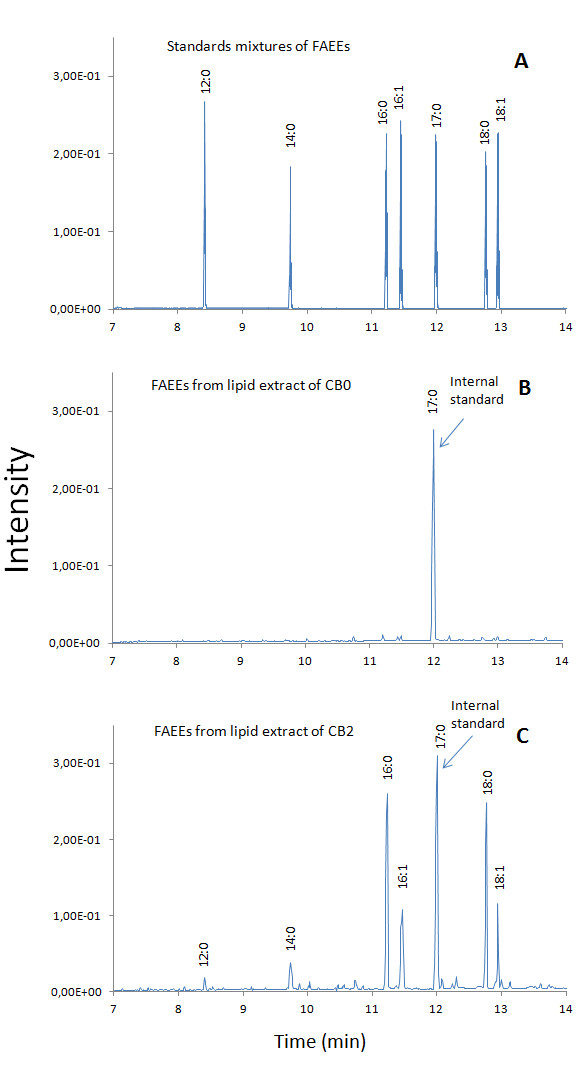
**Gas chromatography-mass spectrometry analysis of fatty acid ethyl esters in lipid extracts from strains CB0 and CB2**. Cells were cultured in SD medium lacking uracil containing 2% glucose. Neutral lipids were purified from total lipid extracts of lyophilized cells by preparative TLC and subjected to GC-MS analyses. The corresponding mass spectra of FAEEs are shown in Additional file 6. **(A) **Gas chromatograms of FAEE standards: 1. lauric acid ethyl ester [12:0]; 2. myristic acid ethyl ester [14:0]; 3. palmitic acid ethyl ester [16:0]; 4. palmitoleic acid ethyl ester [16:1]; 5. heptadecanoic acid ethyl ester [17:0]; 6. octadecanoic acid ethyl ester [18:0]; 7. oleic acid ethyl ester [18:1]. **(B) **Gas chromatogram of FAEEs from lipid extract of CB0 containing 25 μg internal standard, heptadecanoic acid ethyl ester [17:0]. **(C) **Gas chromatogram of FAEEs from lipid extract of CB2 containing 25 μg internal standard, heptadecanoic acid ethyl ester [17:0].

To determine biodiesel formation, all cultures were harvested at the early stationary phase. No significant amounts of extracellular FAEEs were detected in any of the cultures (data not shown). Intracellular lipids were extracted from WS-expressing *S. cerevisiae *and the control strains, separated by thin-layer chromatography (TLC) and analyzed by gas chromatography-mass spectrometry (GC-MS). No FAEEs were detected in the negative control strain *S. cerevisiae *CB0 even when the culture medium was supplemented with 0.1% (w/v) free fatty acids (data not shown). In contrast, biodiesel was formed in WS-expressing strains cultured in SD medium, indicating that WS was responsible for the synthesis of biodiesel (Table 2). The observed mixture of FAEEs was composed of lauric acid ethyl ester (C_14_H_28_O_2_), myristic acid ethyl ester (C_16_H_32_O_2_), palmitic acid ethyl ester (C_18_H_36_O_2_), palmitoleic acid ethyl ester (C_18_H_34_O_2_), octadecanoic acid ethyl ester (C_20_H_40_O_2_) and oleic acid ethyl ester (C_20_H_38_O_2_), the latter four being the main FAEEs (> 95%), which is consistent with the corresponding fatty acids being the dominant ones in yeast [12]. Gas chromatograms are shown in Figure 2 and Additional File 6, using CB0 and CB2 as examples.

**Table 2:** Physiological features and biodiesel production in the wax ester synthase-expressing and reference strains

	CB0	CB1	CB2	CB3	CB4	CB5
Specific growth rate (/h)	0.44 ± 0.01	0.32 ± 0.01	0.36 ± 0.02	0.34 ± 0.01	0.37 ± 0.01	0.33 ± 0.02
q_ethanol _(mmol/g/h)	43 ± 3	28 ± 3	40 ± 4	38 ± 3	36 ± 4	38 ± 4
Ethanol (g/L)^a^	6.9 ± 0.2	5.9 ± 0.2	6.6 ± 0.3	6.5 ± 0.2	6.2 ± 0.3	4.9 ± 0.2
FAEEs (mg/L)	nd	5.0 ± 0.8	6.3 ± 1.2	2.1 ± 0.3	1.3 ± 0.2	2.3 ± 0.4

All strains were cultured in SD medium lacking uracil and containing 2% glucose. Values are the mean of at least three independent assays ± standard deviation. ^a^maximum concentration. FAEE: fatty acid ethyl esters; nd: not detected

In the first part of this study, it was demonstrated that WSs have varied activity for a broad range of linear fatty alcohols, including ethanol. The information on specificities of different WSs suggests that the WS from *M. hydrocarbonoclasticus*, which has the highest relative activity towards ethanol *in vitro*, would also result in the highest biodiesel production. This was confirmed by the results of the FAEE production. As expected, *S. cerevisiae *CB2 expressing WS from *M. hydrocarbonoclasticus *DSM 8798 could produce FAEEs (biodiesel) at a titer of 6.3 mg/L, which is higher than that of any of the other WS-expressing yeast strains (Table 2). The WS from *M. hydrocarbonoclasticus *should therefore be the choice for constructing a biodiesel producing yeast. Additionally, for most of the WS enzymes, DGAT activity has been observed, which results in the formation of TAG, the predominant storage lipid. A previous report showed that WS from *M. hydrocarbonoclasticus *DSM 8798 does not have DGAT activity [21]. This property makes it even more suitable for producing biodiesel as it avoids direction of fatty acyl-CoAs towards TAG biosynthesis.

Growth of WS-expressing strains was performed in SD medium lacking uracil and some physiological features are listed in Table 2. The shake-flask experiments proved that all WS-expressing strains displayed the normal diauxic growth pattern. Initially, glucose was consumed, and once the glucose was exhausted, respiration of ethanol resulted in yet another phase of biomass formation. During the growth on glucose, ethanol is accumulated at a high concentration and hence biodiesel production is not limited by ethanol supply; it is most likely that the supply of fatty acyl-CoAs naturally occurring in *S. cerevisiae *is not sufficient to support biodiesel production at significant amounts.

Acc1p catalyzes the formation of malonyl-CoA, which is the first and crucial step for fatty acid biosynthesis [11], and it has previously been shown that overexpression results in improved production of malonyl-CoA derived products [11,12,28]. The sources of malonyl-CoA are generally supposed to be limited, impeding its utility for overproducing fatty acids [12,28]. We therefore overexpressed Acc1p to enhance the supply of malonyl-CoA in *S. cerevisiae *CB2. As a result, the level of biodiesel produced increased about 30%, resulting in a biodiesel titer of 8.2 ± 1.1 mg/L. This clearly shows that it is possible to direct more flux towards biodiesel in yeast expressing WS and this provides interesting prospects for future metabolic engineering of lipid metabolism in order to further enhance biodiesel production through yeast-based fermentation.

## Conclusions

We have functionally expressed and characterized five WSs from different species in *S. cerevisiae *thus constructing cell factories for production of wax esters. The preferred *in vitro *substrates for the five WS enzyme were identified, and this can be used as a guide to generate specific ester-producing cell factories. Besides the adopted WS, the chain-length compositions of the produced esters are also governed by substrate availability. Additional enzymes and corresponding genes might therefore have to be introduced to ensure *de novo *synthesis of the required substrates. For example, since *S. cerevisiae *is not capable of providing long-chain fatty alcohols as substrates, the conversion of fatty acids to fatty alcohols catalyzed by alcohol-forming fatty acyl reductases is required to form *de novo *wax esters with long-chain alcohols and long-chain acyl-CoAs.

We have used the information of substrate preferences of WSs to produce biodiesel (FAEEs), which requires a WS with activity on ethanol and long-chain fatty acids. WS from *M. hydrocarbonoclasticus *showed the highest preference for ethanol compared to the other WSs, and could permit the engineered *S. cerevisiae *to produce biodiesel to a concentration of 6.3 mg/L. We over-expressed acetyl-CoA carboxylase to further enhance biodiesel production, and this resulted in a 30% increase of the titer. Clearly much more work is needed in order to reach commercial targets of biodiesel, but we are confident that our work provides the basis for advancing towards microbial-based production of biodiesel.

## Methods

### Strains and plasmids

*S. cerevisiae *strains used in this work are listed in Table 3. All DNA manipulations were carried out in *E. coli *DH5α as described by Sambrook and Russell [29]. Plasmids and strains were constructed by the following procedures.

**Table 3:** List of strains used in this study and their genotypes

Strain	Genotype	Plasmid	Source
***E. coli *strains**			
*E. coli *DH5α	*supE*44 *lacU*169 (ϕ80*lacZ *ΔM15) *hsdR*17 *recA*1 *endA*1 *gyrA*96 *thi*-1 *relA*1		
***S. cerevisiae *strains**			
CEN.PK 113-5D	*MAT*a *MAL2-8*c *SUC2 ura3-52*		P. Kötter, University of Frankfurt, Germany
CB0	CEN.PK 113-5D	pSP-GM2	This study
CB1	CEN.PK 113-5D	pSP-GM2 carrying WS gene from *A. baylyi *ADP1	This study
CB2	CEN.PK 113-5D	pSP-GM2 carrying WS gene from *M. hydrocarbonoclasticus *DSM 8798	This study
CB3	CEN.PK 113-5D	pSP-GM2 carrying WS gene from *R. opacus *PD630	This study
CB4	CEN.PK 113-5D	pSP-GM2 carrying WS gene from *Mus musculus *C57BL/6	This study
CB5	CEN.PK 113-5D	pSP-GM2 carrying WS gene from *P. arcticus *273-4	This study
CB2A	CEN.PK 113-5D	pSP-GM2 carrying WS gene from *M. hydrocarbonoclasticus *DSM 8798 and *ACC1*	This study

WS: wax ester synthase

The five sequences of the WS genes from *A. baylyi *ADP1, *M. hydrocarbonoclasticus *DSM 8798, *R. opacus *PD630, *Mus musculus *C57BL/6, and *P. arcticus *273-4 were codon optimized for expression in *S. cerevisiae*. They were synthesized and provided by DNA2.0 (Menlo Park, CA, USA). The gene sequences are listed in Additional files 1, 2, 3, 4 and 5. The genes were amplified by PCR using Phusion DNA polymerase (Finnzymes, Vantaa, Finland) and the oligonucleotide primers listed in Table 4 and restricted with *Bam*HI/*Hin*dIII. The five *Bam*HI/*Hin*dIII digested DNA fragments were, respectively, ligated into vector pSP-GM2 [25] downstream of the constitutively active *TEF1 *promoter. These plasmids were introduced into *S. cerevisiae *CEN.PK113-5D (*MAT a ura3-52 MAL2-8c SUC2*) kindly provided by P. Kötter (University of Frankfurt, Germany) [30] to generate strains CB0 to CB5. The gene encoding Acc1p was amplified using the primers listed in Table 4. The PCR fragment was digested with *Not*I/*Sac*I and ligated to the previously constructed plasmid containing the wax ester synthase gene from *M. hydrocarbonoclasticus *DSM 8798. *ACC1 *was integrated downstream of the *PGK1 *promoter and the resulting plasmid also transformed into *S. cerevisiae *CEN.PK113-5D. Yeast transformation was conducted using the standard LiAc/SS carrier DNA/PEG method [31]. Transformants were selected and obtained on plates of SD medium lacking uracil [32].

**Table 4:** List of primers used in this study

Gene product	Primer sequence 5'→3'	
	
	forward	reverse
Wax ester synthase from *A. baylyi *ADP1 [GenBank: AF529086]	CGGGATCCCGCTCGAGATGCGTCCATT	GGGGTACCCCAAGCTTGGGTTAGTTTGCAG
Wax ester synthase from *M. hydrocarbonoclasticus *DSM 8798 (EF219377)	CGGGATCCCGCTCGAGATGAAGAGATTAGG	GGGGTACCCCAAGCTTGGGTTACTTTCTAGTACG
Wax ester synthase from *R. opacus *PD630 (GQ923886)	CGGGATCCCGCTCGAGTTGACCGACGTGATTAC	GGGGTACCCCAAGCTTGGGTTAGCTAGCCACCACC
Wax ester synthase from *Mus musculus *C57BL/6 (AY611032)	CGGGATCCCGCTCGAGATGTTCTGGCCAACC	GGGGTACCCCAAGCTTGGGTTAAACAATGACCAAC
Wax ester synthase from *P. arcticus *273-4 (YP_263530)	CGGGATCCCGCTCGAGATGAGATTACTGACCGCTGT	GGGGTACCCCAAGCTTGGGTTAAGGGGCCAACT
Acetyl-CoA carboxylase [GenBank: NM_001183193] from *S. cerevisiae*	ATTTGCGGCCGCTTTAGTTTCTACCATGAGCGAAG	GGCGAGCTCGCAAGGTTTATTTCAAAGTCTTC

### Growth conditions

*E. coli *recombinant cells were grown in Luria-Bertani medium in the presence of ampicillin (100 mg/L) at 37°C.

Recombinant strains of *S. cerevisiae *were cultured in 500 mL shake flasks containing 100 mL SD medium lacking uracil and 2% (w/v) glucose at 30°C with reciprocal shaking at 120 rpm, which were inoculated to an optical density at 600 nm of 0.02 from pre-cultures.

### Analytical methods

The growth was measured by optical density at 600 nm and samples were taken in the early stationary phase. The dry cell weight was determined by filtering 5 ml of the cell culture through a 0.45 μm pore-size nitrocellulose filter (Sartorius Stedim, Göttingen, Germany) and measuring the increased weight of the dry filter. The exponential growth rate was calculated by log-linear regression analysis of the biomass versus cultivation time. The concentrations of residual glucose and external metabolites were analyzed using the Dionex Ultimate 3000 HPLC system (Dionex Softron GmbH, Germering, Germany) with an Aminex HPX-87H column (Bio-Rad, CA, USA) using 5 mM H_2_SO_4 _as the mobile phase.

### Lipid extraction and thin-layer chromatography

The harvested cells were washed twice with distilled water and freeze-dried for about three days or until they appeared dry. Lipids were extracted from the lyophilized cell pellets using the previously reported method [33]; 25 μL of heptadecanoic acid ethyl ester was used as the internal standard. FAEEs in the total lipid extracts were purified by preparative TLC [9] using TLC Silica gel 60 F254plates (Merck, Darmstadt, Germany) and the solvent system of heptane, 2-propanol and acetic acid in the ratio 95:5:1 (v/v/v). Lipids were visualized by being sprayed with 0.05% 2,7-dichlorofluoresceine in ethanol. Heptadecanoic acid, glyceryl triheptadecanoate, cholesteryl palmitate and heptadecanoic acid ethyl ester (Sigma-Aldrich, St. Louis, MO, USA) were used as reference substances for free fatty acids, TAGs, sterol esters and FAEEs, respectively. The spots corresponding to FAEEs were scraped from the TLC plate using a razor-blade and transferred to 12-mL Teflon-lined screw-capped tubes. FAEEs were extracted from the scraped TLC powder with 7 mL of a hexane, methanol and water mixture (3:2:2). After centrifuging for 5 min at 3000 relative centrifugal force, an aliquot of the upper phase was transferred to a GC-vial and used for GC-MS analysis.

### Gas chromatography-mass spectrometry analysis of fatty acid ethyl esters

The FAEEs were separated and quantified using a Focus GC DSQ II single quadruple GC-MS (Thermo Fisher Scientific, Waltham, MA, USA). The separation was performed on a Zebron (ZB-WAX) GC column (30 m × 0.25 mm internal diameter, 0.25 μm film thickness; Phenomenex, Macclesfield, UK). A 1-μL portion of the organic phase was injected into the GC-MS using splitless injection (1 μL at 250°C); helium was used as a carrier gas (1 μL/min). The chromatographic separation was initially set at 50°C (1.5 min), then the temperature was increased to 180°C (25°C per min), and finally the temperature was increased to 250°C (10°C per min) and held for 3 min. The mass transfer line and ion source were at 250°C and 200°C, respectively. The FAEEs were detected with electron ionization (70 eV) in scan mode (50 to 650 *m/z*) and selected ion monitoring mode at *m/z *88 and 55 (for quantitative analysis).

The identification of unknown FAEEs was achieved by comparison of their retention times and mass spectrum profiles with known standards (Cayman Chemical, Ann Arbor, MI, USA). The standards used in this study were lauric acid ethyl ester, myristic acid ethyl ester, palmitic acid ethyl ester, palmitoleic acid ethyl ester, heptadecanoic acid ethyl ester, stearic acid ethyl ester and oleic acid ethyl ester. The quantification of FAEEs was performed using the QuanBrowser function in Xcalibur software version 2.0 (Thermo Fisher Scientific).

### Enzyme activity assay

Cell-free extracts were prepared using a previously reported fast preparation method for enzyme analysis [34]. WS activities in the extracts were measured *in vitro *using [1-^14^C] palmitoyl-CoA and alcohols with varied chain length (C_2 _to C_16_) as substrates [9].

## Abbreviations

Acc1p: acetyl-CoA carboxylase; acyl-CoA: fatty acyl-coenzyme A; CoA: coenzyme A; DGAT: acyl-CoA:diacylglycerol acyltransferase; FAEE: fatty acid ethyl ester; GC-MS: gas chromatography-mass spectrometry; HEK: human embryonic kidney cells; OD: optical density; PEG: poly(ethylene) glycol; SD: synthetic minimal dropout medium; TAG: triacylglycerol; TLC: thin-layer chromatography; WS: wax ester synthase.

## Competing interests

The authors declare that they have no competing interests.

## Authors' contributions

SS, VS and JN designed and coordinated this work. SS and JV performed the experiments. SK established the method for GC-MS analysis of FAEEs. SS performed the data analysis and wrote the manuscript. All authors read and approved the final manuscript.

## Supplementary Material

Additional file 6 **Figure S1, mass spectra of FAEE standards derived from GC-MS analysis**. Mass spectra correspond to FAEEs in Figure 2: **(A) **lauric acid ethyl ester [12:0]; **(B) **myristic acid ethyl ester [14:0]; **(C) **palmitic acid ethyl ester [16:0]; **(D) **palmitoleic acid ethyl ester [16:1]; **(E) **heptadecanoic acid ethyl ester [17:0]; **(F) **octadecanoic acid ethyl ester [18:0]; **(G) **oleic acid ethyl ester [18:1]. Click here for file

Additional file 1 **Table S1, sequence of wax ester synthase gene from *Acinetobacter baylyi *ADP1 codon optimized for expression in a yeast host**. Click here for file

Additional file 2 **Table S2, sequence of wax ester synthase gene from *Marinobacter hydrocarbonoclasticus *DSM 8798 codon optimized for expression in a yeast host**. Click here for file

Additional file 3 **Table S3, sequence of wax ester synthase gene from *Rhodococcus opacus *PD630 codon optimized for expression in a yeast host**. Click here for file

Additional file 4 **Table S4, sequence of wax ester synthase gene from *Mus musculus *C57BL/6 codon optimized for expression in a yeast host**. Click here for file

Additional file 5 **Table S5, sequence of ester synthase from *Psychrobacter articus *273-4 codon optimized wax for expression in a yeast host**. Click here for file
