# Supplementary figures and images for: Functional expression and characterization of five wax ester synthases in Saccharomyces cerevisiae and their utility for biodiesel production
Source: Biotechnol Biofuels. 2012 Feb 24;5:7. doi: 10.1186/1754-6834-5-7 (PMC3309958; doi:10.1186/1754-6834-5-7)

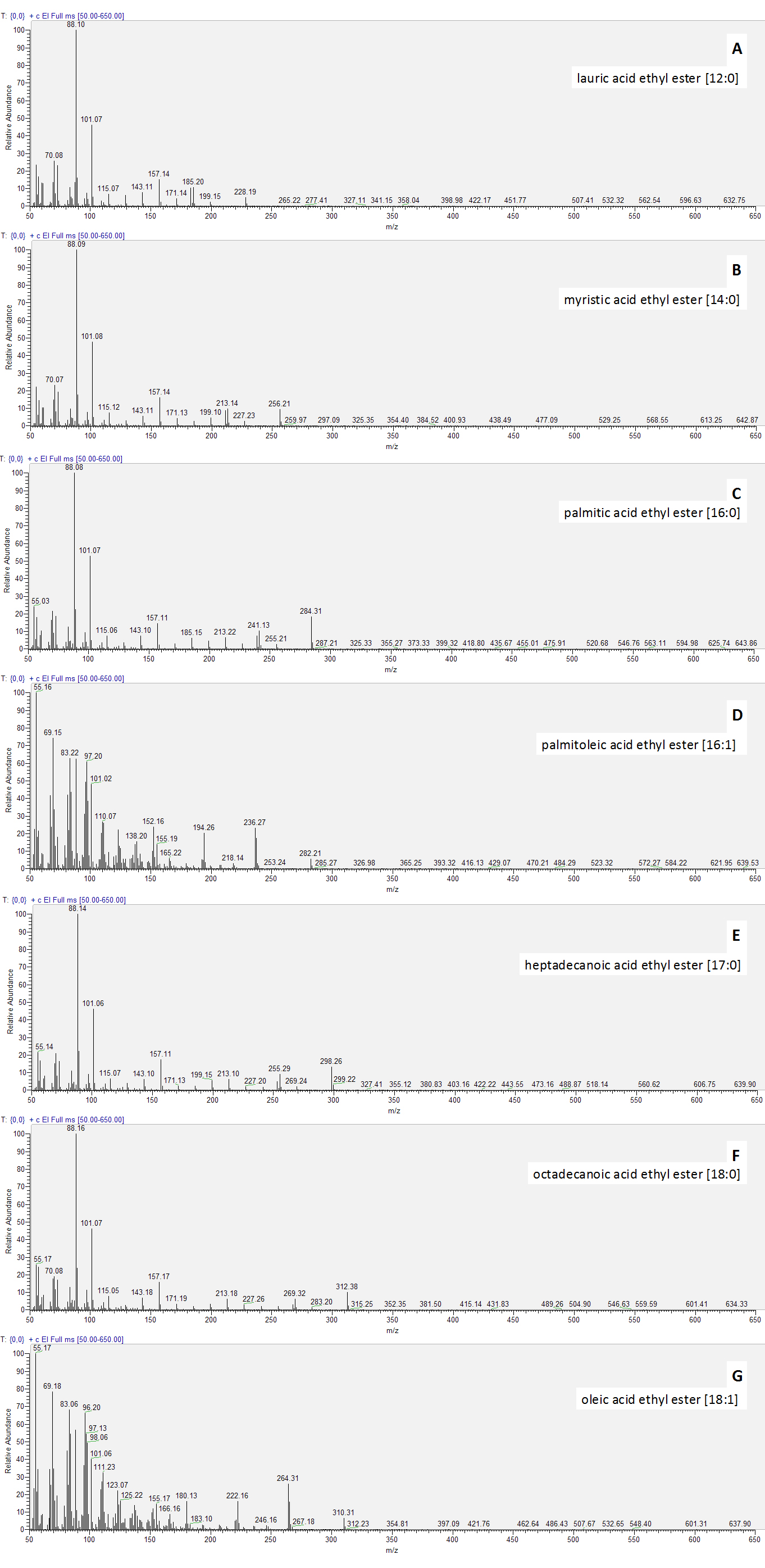

Supplement: Additional file 6 — Figure S1, mass spectra of FAEE standards derived from GC-MS analysis. Mass spectra correspond to FAEEs in Figure 2: (A) lauric acid ethyl ester [12:0]; (B) myristic acid ethyl ester [14:0]; (C) palmitic acid ethyl ester [16:0]; (D) palmitoleic acid ethyl ester [16:1]; (E) heptadecanoic acid ethyl ester [17:0]; (F) octadecanoic acid ethyl ester [18:0]; (G) oleic acid ethyl ester [18:1]. [file 1754-6834-5-7-S6.JPEG]
