# Supplementary material for: Functional expression and characterization of five wax ester synthases in Saccharomyces cerevisiae and their utility for biodiesel production
Source: Biotechnol Biofuels. 2012 Feb 24;5:7. doi: 10.1186/1754-6834-5-7 (PMC3309958; doi:10.1186/1754-6834-5-7)
Supplement: Additional file 1 — Table S1, sequence of wax ester synthase gene from Acinetobacter baylyi ADP1 codon optimized for expression in a yeast host. [file 1754-6834-5-7-S1.DOC]

**Additional file 1**

**Table S1 Sequence of wax ester synthase gene from *Acinetobacter baylyi* ADP1 codon optimized for expression in a yeast host. The following sequence was synthesized and provided by DNA2.0 (Menlo Park, CA, USA)**

| Name | Optimized wax ester synthase gene from *Acinetobacter baylyi* ADP1 |
| --- | --- |
| Sequence | ATGCGTCCATTACATCCAATTGATTTCATCTTCCTATCTTTAGAAAAGAGACAGCAACCAATGCATGTAGGCGGGCTTTTTCTCTTCCAAATTCCTGACAATGCTCCAGACACATTCATCCAAGACTTGGTTAATGATATCAGAATTTCCAAGTCAATACCTGTTCCACCTTTCAATAACAAACTCAACGGCTTGTTCTGGGACGAGGATGAAGAGTTCGATCTGGATCATCACTTTCGACATATCGCATTACCACACCCTGGTAGAATCAGGGAGTTACTCATCTACATTTCCCAAGAGCATTCAACATTACTTGATAGAGCCAAGCCATTGTGGACATGCAACATAATCGAAGGTATAGAAGGCAATAGATTTGCCATGTACTTCAAGATACATCACGCTATGGTTGATGGAGTCGCTGGAATGAGACTAATCGAGAAGAGCCTTTCTCACGATGTTACAGAAAAGTCAATTGTACCACCTTGGTGTGTAGAAGGTAAACGAGCTAAGCGTTTACGTGAACCAAAAACCGGAAAGATCAAAAAGATTATGTCTGGTATCAAATCTCAACTTCAAGCCACGCCTACTGTCATTCAAGAGTTGTCTCAAACAGTGTTCAAAGACATTGGTAGAAACCCAGATCACGTGTCCTCATTTCAAGCGCCATGTTCTATTCTGAACCAAAGAGTATCCAGTAGTAGAAGATTTGCAGCACAGTCTTTTGATCTTGATAGGTTCAGAAACATTGCAAAGTCTCTGAACGTCACCATAAACGACGTGGTTCTAGCTGTTTGCTCTGGGGCACTGAGAGCTTATCTAATGTCACATAACAGCTTGCCATCAAAACCATTGATTGCGATGGTTCCTGCCTCTATACGTAATGATGATTCAGATGTCAGTAACAGAATAACAATGATCCTTGCCAACCTAGCTACTCATAAGGATGATCCTTTGCAGAGATTAGAAATCATTAGAAGATCAGTGCAAAACTCAAAGCAGAGATTCAAAAGGATGACCAGTGATCAAATCTTGAATTACTCTGCAGTGGTATACGGTCCAGCTGGTCTGAATATCATATCAGGAATGATGCCAAAAAGACAAGCCTTTAACTTAGTTATCTCCAATGTACCTGGTCCACGAGAACCTCTCTACTGGAACGGAGCTAAGTTGGATGCACTTTACCCAGCCTCTATCGTTTTAGATGGTCAGGCTTTGAACATTACAATGACTAGTTATCTAGACAAGCTAGAAGTTGGGTTGATTGCGTGTAGAAATGCCCTACCTAGAATGCAGAATTTGCTGACTCACTTAGAAGAGGAGATTCAACTCTTTGAAGGCGTCATCGCAAAACAAGAGGATATCAAAACTGCAAACTAA |
