# Supplementary material for: Functional expression and characterization of five wax ester synthases in Saccharomyces cerevisiae and their utility for biodiesel production
Source: Biotechnol Biofuels. 2012 Feb 24;5:7. doi: 10.1186/1754-6834-5-7 (PMC3309958; doi:10.1186/1754-6834-5-7)
Supplement: Additional file 2 — Table S2, sequence of wax ester synthase gene from Marinobacter hydrocarbonoclasticus DSM 8798 codon optimized for expression in a yeast host. [file 1754-6834-5-7-S2.DOC]

**Additional file 2**

**Table S2 Sequence of wax ester synthase gene from *Marinobacter hydrocarbonoclasticus* DSM 8798 codon optimized for expression in a yeast host. The following sequence was synthesized and provided by DNA2.0 (Menlo Park, CA, USA)**

| Name | Optimized wax ester synthase gene from *Marinobacter hydrocarbonoclasticus* DSM 8798 |
| --- | --- |
| Sequence | ATGAAGAGATTAGGTACTCTAGACGCTAGTTGGCTTGCAGTCGAATCCGAAGATACGCCAATGCACGTGGGCACTCTCCAAATCTTCTCATTACCAGAAGGTGCTCCAGAGACATTTCTACGTGATATGGTTACAAGGATGAAAGAGGCAGGAGATGTTGCCCCACCATGGGGTTACAAGCTCGCATGGTCCGGTTTCCTTGGCAGGGTTATTGCTCCTGCCTGGAAGG TAGACAAAGATATCGATTTGGATTATCATGTCCGACATAGTGCATTGCCAAGACCAGGTGGTGAAAGAGAGCTAGGGATACTTGTTTCTAGATTACACTCCAACCCTTTAGATTTCTCTAGACCACTATGGGAATGCCATGTCATTGAAGGTCTTGAAAACAACAGATTTGCACTGTATACTAAGATGCATCACTCTATGATTGATGGGATATCTGGAGTAAGATTGATGCAAAGAGTATTGACCACTGACCCAGAGAGATGTAACATGCCTCCTCCATGGACAGTTAGACCTCACCAGAGAAGAGGAGCTAAAACAGATAAAGAGGCTTCTGTGCCTGCTGCGGTTTCTCAAGCAATGGACGCCTTGAAGCTCCAAGCGGATATGGCCCCTAGACTATGGCAAGCTGGCAATCGTCTAGTACATTCTGTCAGACACCCTGAGGATGGCTTAACAGCTCCATTCACCGGTCCAGTGTCTGTCCTTAACCATAGAGTTACAGCGCAGAGAAGATTCGCTACTCAACACTACCAACTAGATAGATTGAAAAACTTAGCGCATGCCAGTGGTGGTTCACTGAATGATATAGTGCTTTACTTATGTGGTACTGCCTTGAGAAGGTTTTTGGCTGAGCAGAATAACTTGCCTGACACACCTTTAACGGCAGGAATTCCAGTGAATATCAGACCAGCTGATGACGAAGGCACCGGAACACAAATCTCATTCATGATTGCTAGTTTGGCTACTGACGAAGCTGATCCTCTCAATAGATTACAACAGATCAAAACCTCAACACGAAGGGCGAAGGAGCATCTCCAAAAGTTGCCTAAGTCAGCACTAACACAATACACAATGCTGCTGATGTCACCTTACATCTTACAATTGATGAGCGGATTGGGAGGTAGAATGAGGCCAGTTTTCAATGTTACTATAAGCAATGTCCCTGGGCCTGAGGGGACATTGTATTACGAAGGAGCTAGATTGGAAGCCATGTACCCAGTTTCCCTTATCGCCCACGGTGGTGCCTTGAACATCACATGCCTGTCTTACGCTGGCTCCCTTAACTTTGGGTTTACCGGTTGTCGTGATACTTTACCATCAATGCAAAAGTTAGCAGTCTATACTGGTGAAGCATTGGATGAACTCGAATCTCTAATTCTGCCACCAAAGAAGCGTGCCCGTACTAGAAAGTAA |
